# The Ontogeny of Cytochrome P450 Enzyme Activity and Protein Abundance in Conventional Pigs in Support of Preclinical Pediatric Drug Research

**DOI:** 10.3389/fphar.2018.00470

**Published:** 2018-05-14

**Authors:** Joske Millecam, Laura De Clerck, Elisabeth Govaert, Mathias Devreese, Elke Gasthuys, Wim Schelstraete, Dieter Deforce, Lies De Bock, Jan Van Bocxlaer, Stanislas Sys, Siska Croubels

**Affiliations:** ^1^Laboratory of Pharmacology and Toxicology, Department of Pharmacology, Toxicology and Biochemistry, Faculty of Veterinary Medicine, Ghent University, Merelbeke, Belgium; ^2^Laboratory of Pharmaceutical Biotechnology, Department of Pharmaceutics, Faculty of Pharmaceutical Sciences, Ghent University, Ghent, Belgium; ^3^Laboratory of Medical Biochemistry and Clinical Analysis, Department of Bioanalysis, Faculty of Pharmaceutical Sciences, Ghent University, Ghent, Belgium; ^4^Department of Internal Medicine and Clinical Biology of Large Animals, Faculty of Veterinary Medicine, Ghent University, Merelbeke, Belgium

**Keywords:** cytochrome P450, ontogeny, MPPGL, pig, proteomics, enzyme activity, intrinsic clearance

## Abstract

Since the implementation of several legislations to improve pediatric drug research, more pediatric clinical trials are being performed. In order to optimize these pediatric trials, adequate preclinical data are necessary, which are usually obtained by juvenile animal models. The growing piglet has been increasingly suggested as a potential animal model due to a high degree of anatomical and physiological similarities with humans. However, physiological data in pigs on the ontogeny of major organs involved in absorption, distribution, metabolism, and excretion of drugs are largely lacking. The aim of this study was to unravel the ontogeny of porcine hepatic drug metabolizing cytochrome P450 enzyme (CYP450) activities as well as protein abundances. Liver microsomes from 16 conventional pigs (8 males and 8 females) per age group: 2 days, 4 weeks, 8 weeks, and 6–7 months were prepared. Activity measurements were performed with substrates of major human CYP450 enzymes: midazolam (CYP3A), tolbutamide (CYP2C), and chlorzoxazone (CYP2E). Next, the hepatic scaling factor, microsomal protein per gram liver (MPPGL), was determined to correct for enzyme losses during the fractionation process. Finally, protein abundance was determined using proteomics and correlated with enzyme activity. No significant sex differences within each age category were observed in enzyme activity or MPPGL. The biotransformation rate of all three substrates increased with age, comparable with human maturation of CYP450 enzymes. The MPPGL decreased from birth till 8 weeks of age followed by an increase till 6–7 months of age. Significant sex differences in protein abundance were observed for CYP1A2, CYP2A19, CYP3A22, CYP4V2, CYP2C36, CYP2E_1, and CYP2E_2. Midazolam and tolbutamide are considered good substrates to evaluate porcine CYP3A/2C enzymes, respectively. However, chlorzoxazone is not advised to evaluate porcine CYP2E enzyme activity. The increase in biotransformation rate with age can be attributed to an increase in absolute amount of CYP450 proteins. Finally, developmental changes were observed regarding the involvement of specific CYP450 enzymes in the biotransformation of the different substrates.

## Introduction

It has been about 20 years since the first pediatric legislations were initiated by the EMA and the FDA to stimulate pediatric drug research. Over the years this has led to an increased number of pediatric clinical trials, new medicines and pediatric indications both in the European Union and the United States (Califf, 2016; EMA, 2016). In order to support the safety of these pediatric trials, it is important to improve and optimize pediatric preclinical studies. Pediatric juvenile animal studies can provide essential information regarding the PK, PD and safety of a drug. According to the ICH guideline M3(R2), these studies should only be considered when previous animal data and human safety data are insufficient to support pediatric studies. If a juvenile study is warranted, only one relevant species, preferably rodent, is generally considered adequate. This is in contrast with preclinical studies in adults where both rodent and non-rodent species are mandatory (Food and Drug Administration, HHS, 2010). When selecting the appropriate animal species, it is important to take the maturation and growth of the different organ systems into account. From a historical point of view the rat is still the species of first choice when performing pediatric preclinical studies (Andrew et al., 2013). However, more recent reports suggest the pig (*Sus scrofa domestica)* as a valuable alternative animal model to the rat (Bode et al., 2010; Swindle et al., 2012; Helke and Swindle, 2013). Gasthuys et al. (2016) reported a high level of anatomical and physiological similarities between conventional pigs and humans during the developmental stages of life, rendering the piglet a suitable juvenile animal model. Nevertheless still a lot of knowledge gaps remain, such as the maturation of ADME processes both in growing children and piglets.

CYP450 enzymes are the most important phase I drug metabolizing enzymes, located on the smooth endoplasmic reticulum and highly expressed in liver compared to other tissues in pigs (Antonovic and Martinez, 2011; Nielsen et al., 2017). They metabolize between 70 and 80% of the human drugs used, most frequently by hydroxylation (Matalová et al., 2016). The ontogeny of the human CYP450 enzymes is rather well known. For example CYP2C9 enzyme activity increases rapidly after birth reaching adult values around 3 months of age, whereas CYP1A2 reaches adult values around 10 years of age (Alcorn and McNamara, 2002; Johnson et al., 2006; Matalová et al., 2016). In contrast to men, the ontogeny of CYP450 enzymes in pigs remains largely unknown. Hu (2015) reported in Camborough-29 pigs an increase in average total CYP450 contents in liver microsomes and in biotransformation rate with age for six different substrates (phenacetin, coumarin, tolbutamide, bufuralol, chlorzoxazone, and midazolam) (Hu, 2015). Postnatal maturation of CYP450 enzyme activity in liver microsomes of the Göttingen minipig using four different substrates (phenacetin, tolbutamide, midazolam, and dextromethorphan) was reported by Van Peer et al. (2017). In general, CYP3A4, CYP2C9, and CYP2E1 are estimated to contribute to the metabolism of over 50% of human drugs currently on the market (Alcorn and McNamara, 2002). There is evidence that the three probe substrates, midazolam, tolbutamide, and chlorzoxazone, which are often used in the pharmaceutical industry for human *in vitro* work, are biotransformed to the same metabolites in pigs as in humans (Yuan et al., 2002; Helke and Swindle, 2013). The 1-hydroxylation of midazolam, 4-hydroxylation of tolbutamide, and 6-hydroxylation of chlorzoxazone have been used before as markers for, respectively, CYP3A, CYP2C, and CYP2E in pigs (Hu, 2015; Van Peer et al., 2017). Although high percentages of sequence identity of nucleotides and amino acids between pigs and humans are present, this is not always reflected in the substrate affinities (Puccinelli et al., 2011). For example, tolbutamide which is a selective substrate for human CYP2C9 shows some cross-reactivity in pigs, indicating that either the substrate is not specific or other enzymes are involved in the biotransformation (Skaanild, 2006).

When using microsomes to examine the biotransformation of drugs, the final goal is determining the intrinsic clearance and extrapolation to *in vivo* (IVIVE). This can be done by using hepatic scaling factors such as the MPPGL. In men, the MPPGL increases from birth (26 mg/g) to a maximum of 40 mg/g at approximately 28 years of age, followed by a gradual decrease with aging (Barter et al., 2007, 2008). In pigs, this has only been done in two Suffolk White adult pigs obtaining values of 32.6 and 36.2 mg/g liver (Achour et al., 2011). Finally, the intrinsic clearance can be calculated with liver weight as a second scaling factor (Barter et al., 2007).

Another important factor in IVIVE is the CYP450 protein abundance. Barter et al. (2013) integrated specific abundances and phenotypes associated with several CYP450s in Caucasian and Chinese population models. This led to simulation of observed population differences in the clearance of several reference drugs. Regarding the pediatric population, and especially children younger than 2 years of age, Johnson et al. (2006) showed that physiologically based models resulted in better predictions than simple allometric scaling. The CYP450 protein abundances can thus support a better quantitative understanding of species differences and result in better scaling of PK and safety data to the clinical setting.

The aim of the current study was to gain more insight in the ontogeny of porcine CYP450 enzymes. First, the CYP450 enzyme activity was determined in hepatic microsomes using three probe substrates (midazolam, tolbutamide, and chlorzoxazone) in four age categories of growing piglets. Next to activity measurements, the MPPGL was determined in order to calculate the intrinsic clearance. Finally, the protein abundance of hepatic CYP450 in the microsomes was measured and compared with the enzyme activity.

## Materials and Methods

### Chemicals

The following chemicals were purchased from VWR (Oud-Heverlee, Belgium), dipotassium hydrogen phosphate, potassium dihydrogen phosphate, KCL, and glycerol. Tolbutamide (TB), chlorzoxazone (CZ), chlorpropamide, formic acid, cytochrome *c* from equine heart, KCN, TEABC, DTT, MMTS, CaCl_2_, DMSO were purchased from Sigma-Aldrich (St. Louis, MO, United States). Xylazine (Xyl-M^®^ 2%) was obtained from VMD (Arendonk, Belgium). Tiletamine-zolazepam (Zoletil^®^ 100) was purchased from Virbac (Carros, France). Sodium pentobarbital 20%^®^ was obtained from Kela (Hoogstraten, Belgium). The Coomassie Bradford Protein Assay Kit was purchased from Thermo Fisher Scientific (Asse, Belgium). Midazolam (MDZ), 1-hydroxy-midazolam (1-OH-MDZ), 6-hydroxy-chlorzoxazone (6-OH-CZ), and 4-hydroxy-tolbutamide (4-OH-TB) were all obtained from Alsachim (Illkirch-Graffenstaden, France). NADPH was purchased from Gentaur (Kampenhout, Belgium). UPLC-water and ACN were obtained from Biosolve (Valkenswaard, Netherlands). Trypsin was purchased from Promega (Madison, WI, United States). Beta-galactosidase was purchased from Sciex (Framingham, MA, United States). Hi3 *E. coli* was purchased from Waters (Zellik, Belgium). The stock solutions of the probe substrates, the metabolites and the internal standard were prepared separately in methanol (MeOH) at a concentration of 1 mg/mL and stored at -80°C.

### Animals and Sample Collection

The current study was approved by the ethical committee of the Faculties of Veterinary Medicine and Bioscience Engineering of Ghent University (EC2016/105). Care and use of the animals was in full compliance with the national (Belgian Royal Decree of 29 May 2013) and European legislation on animal welfare and ethics (Anonymous, 2010/63/EU). Sixteen pigs (8 barrows and 8 sows, Hybrid sow × Piétrain boar) aged 2 days, 4 weeks, and 8 weeks were purchased from Gesuporc BVBA (Vorselaar, Belgium). Eight intact boars aged 6 months and eight sows aged 7 months (Large white × Land race, Seghers hybrid) were purchased from RA-SE Genetics and Convis (Ettelbruck, Luxembourg). The same strain of Seghers hybrids were used as the mothers of the younger age categories, thus reducing interindividual variability. The 2-day, 4-week, and 8-week males were castrated. However, the mean age for boars to reach puberty was reported to be at 21 weeks of age, while at 26 weeks of age only 8–31% of gilts were in puberty according to Van den Broeke et al. (2015). Therefore, no effect of castration (and thus sex hormones) is expected on the results. The four chosen age categories represent different groups of growing children. Two-day-, four-week-, eight-week-, and six-to-seven-month-old pigs correspond with neonates, infants, children, and adolescents, respectively (Shayne, 2015). The sows were 1 month older than the boars as females reach puberty later compared to males (Van den Broeke et al., 2015). All pigs were weighed (BW, kg) and euthanized at arrival at the test facility by intramuscular injection of a mixture of xylazine and tiletamine-zolazepam, followed by intracardial injection of a sodium pentobarbital overdose. The liver was excised, patted dry with compresses and weighed (LW, g). A piece of the right liver lobe (1.22–8.18 g) was snap-frozen in liquid nitrogen and stored at -80°C until microsome preparation. CYP450 enzymes were stable in frozen liver samples for up to 4 months of storage time (Schelstraete et al., 2018).

### Preparation of Microsomes

Microsomes were prepared as described by Goossens et al. (2013) using the differential ultracentrifugation method (Wilson et al., 2003). In short, liver samples were thawed on ice while soaking in homogenization buffer (0.25 M aqueous phosphate buffer containing 1.15% KCl, pH 7.25). The liver tissue was weighed, minced with a scissor and homogenized in 16 mL of homogenization buffer using a Potter-Elvehjem system (VWR). An aliquot of the homogenate was frozen (-80°C) in order to determine the MPPGL (see section “Microsomal Protein per Gram Liver”). After centrifugation (Beckman L8-70M Ultracentrifuge, Beckman Coulter Limited, High Wycombe, United Kingdom) at 10,000 *g* for 25 min at 4°C, the supernatant was centrifuged at 100,000 *g* for 80 min at 4°C. The microsomal pellet was washed by resuspension in 16 mL of homogenization buffer, followed by centrifuging according to the latter conditions. The final microsomal pellet was resuspended in 1.5 mL/g liver tissue resuspension buffer (0.25 M aqueous phosphate buffer containing 1.15% KCl and 30% glycerol, pH 7.25). The microsomal suspension was snap-frozen in liquid nitrogen and stored at -80°C until further analysis.

### CYP450 Activity Measurements

Activity measurements were performed as described by Goossens et al. (2013). Total protein concentration in the microsomes was determined using the Bradford assay (Bradford, 1976). Three different probe substrates, namely midazolam, chlorzoxazone, and tolbutamide, were used to determine the CYP3A, CYP2E, and CYP2C enzyme activity, respectively. The formation rate of 1-hydroxy-midazolam, 6-hydroxy-chlorzoxazone, and 4-hydroxy-tolbutamide was analyzed, respectively. In order to optimize the ideal incubation conditions, the linearity, K_M_ and maximum biotransformation rate (V_max_) were determined on a pool of four 6 months old pigs (results not shown). An apparent *K*_M_ of 13.7, 126.2, and 613.2 μM for midazolam, chlorzoxazone, and tolbutamide, respectively, was determined. The apparent *V*_max_ was estimated at 281.3, 1210.1, and 153.3 μmol/min/mg microsomal protein, respectively. The incubation conditions were as follows. The preheated (37°C) incubation mixture consisted of 50 μL of 1.15% KCl in water, 50 μL of 0.05 M aqueous phosphate buffer (pH 7.4), and 50 μL of substrate in water mixed with 50 μL NADPH (final concentration of 1 mM). After 3 min of pre-incubation, the enzymatic reaction was initiated by addition of 50 μL of microsome suspension. The microsomes were diluted in a 1.15% KCl solution, resulting in a final concentration of 0.25 mg/mL in the incubation mixture for all substrates. Midazolam (10 μM) was incubated for 10 min, while chlorzoxazone (100 μM) and tolbutamide (100 μM) were incubated for 20 min. After the specified incubation time, the enzymatic reactions were terminated by adding 25 μL of ice cold water/ACN/formic acid mixture [42/55/3, (v/v/v)], containing the internal standard chlorpropamide (final concentration of 0.072 μM). Next, the incubation mixtures were vortexed and transferred on ice. Subsequently, the samples were centrifuged at 20,000 *g* for 10 min (4°C) after which the supernatant was collected and frozen at -20°C until analysis. Each microsomal sample was incubated separately with the three substrates in triplicate. The metabolites formed during these incubations were quantified using a validated UHPLC-MS/MS method (De Bock et al., 2012).

The microsomal activity expressed as pmol/min/mg microsomal protein was corrected to nmol/min/g liver by means of the MPPGL (equation 1; section “Microsomal Protein per Gram Liver”).

Enzyme activity (nmol/min/g liver)=(Microsomal activity (pmol/min/mg microsomal protein)*MPPGL (mg/g liver))/1000⁢      (Eq. 1)

### Microsomal Protein Per Gram Liver

The MPPGL of each pig (2-day-old ♂ = 2, ♀ = 8; 4 weeks, 8 weeks, and 6–7 months each time ♂ = 8, ♀ = 8) was calculated after determination of the microsomal recovery factor. This recovery factor was estimated by analyzing the NCR activity in homogenate and microsomes, according to Guengerich et al. (2009). Homogenate and microsomes were both diluted to 60 mg/mL (100 mg/mL for the 2-day-old piglets). 0.5 mM equine heart cytochrome *c* in 10 mM aqueous potassium phosphate buffer (pH 7.7) was prepared and frozen (-20°C) until use. Nine-hundred microliters of 0.3 M potassium phosphate buffer (pH 7.7) containing 1 mM KCN, 80 μL of cytochrome *c* (0.5 mM) and 10 μL of the diluted homogenate or microsomes were mixed. After addition and mixing of 10 μL of reduced NADPH solution (10 mM in water), the absorbance was monitored at 550 nm for 4 min. The rate of cytochrome *c* reduction or NCR was calculated according to equation 2:

ΔA550/min/0.021=nmol of cytochrome  c  reduced per minutein the cuvette     (Eq. 2)

The microsomal recovery factor was then calculated by the ratio of the NCR in the microsomes and the homogenate. Finally the MPPGL was calculated by the following equation (equation 3):

MPPGL (mg/g)=yield of microsomal protein (mg/g)/microsomal recovery factor     (Eq. 3)

### CYP450 Protein Abundancy Measurement: HD-DDA MS Experimental Set-up

Microsomal proteins (20 μg) of each individual pig were reduced in 0.5 M TEABC and 1 mM DTT for 1 h at 60°C, followed by alkylation using 10 mM MMTS for 10 min at room temperature. Digestion of proteins into peptides was performed using trypsin (33:1 protein/enzyme ratio) overnight at 37°C with CaCl_2_ and ACN to a final concentration of 1 mM and 5%, respectively, as described earlier (Glibert et al., 2014). After evaporation, the samples were resuspended in 0.1% formic acid. Four hundred nanograms sample was spiked with 50 fmol beta-galactosidase and 50 fmol Hi3 *E. coli* standards before injection.

The peptides were separated using a nanoscale UPLC system (nanoACQUITY UPLC^®^, Waters, Milford, MA, United States) coupled to a Synapt G2-Si mass spectrometer (Waters). Peptides were first trapped in 0.1% formic acid on a 180 μm × 20 mm C18 Trap column. Separation was performed on a HSS C18 1.8 μm, 75 μm × 250 mm analytical column at a flow rate of 300 nL/min and a temperature of 45°C. Mobile phase A and B were composed of, respectively, 0.1% formic acid with 4% DMSO in UPLC-water and 80% ACN containing 0.1% formic acid. Peptides were separated with a linear gradient for 60 min at 1–40% solvent B and for 1 min 40–85% solvent B. The Q-TOF Synapt G2-Si instrument was operated in positive mode for High Definition-DDA, using a nano-ESI source, acquiring full scan MS and MS/MS spectra (*m/z* at 50–5,000) in resolution mode. Survey MS scans were acquired using a fixed scan time of 200 ms. Tandem mass spectra of up to eight precursor ions with charge state 2+ to 5+ were generated using CID in the trapping region with intensity threshold set at 3,000 cps, using a collision energy ramp from 6/9 V (low mass, start/end) up to 147/183 V (high mass, start/end). MS/MS scan time was set to 100 ms with an accumulated ion count ‘TIC stop parameter’ of 100,000 cps allowing a maximum accumulation time of 250 ms. Dynamic exclusion of fragmented precursor ions was set to 12 s. Ion mobility wave velocity was ramped from 2,500 to 400 m/s for wideband enhancement to obtain a near-100% duty cycle on singly charged fragment ions. LockSpray of glufibrinopeptide-B (*m/z* at 785.8427) was acquired at a scan frequency of 60 s.

Data analysis of the raw files obtained from the Synapt G2-Si was performed in Progenesis^TM^ QI (Nonlinear Dynamics) version 2.3. Peptides with charge +1 were discarded. For relative quantification, data was normalized to all proteins. For absolute quantification, data was normalized to Hi3 *E. coli* peptides. Peptide identification was performed with Mascot 2.5, the following search criteria were set: trypsin as digestion enzyme, up to two missed cleavages allowed, fixed modification of methylthio cysteine and variable modifications of methionine oxidation and deamidation at asparagine and glutamine. Peptide mass tolerance was set to 15 ppm and fragment mass tolerance to 0.2 Da. Protein identifications were obtained by searching a compiled database of reviewed *Sus scrofa* entries (SwissProt), supplemented with unreviewed CYP proteins and fragments of interest, the cRAP database (laboratory proteins and dust/contact proteins^1^) and sequences of spiked standard proteins. For relative quantification, the top three peptides were used and only proteins with at least one unique peptide were further considered. For absolute quantification, proteins were quantified using the top three unique peptides against Hi3 *E. coli* peptides, and only proteins with at least one unique peptide were further considered. Protein data was exported from Progenesis^TM^ for further statistical analysis.

### Statistical Analysis

Sex and age differences in enzyme activity, MPPGL, and protein abundances were analyzed by a two-way nested ANOVA on the log-transformed data. Homogeneity of variances was evaluated using Levene’s test. Tukey’s honest significant difference test was performed *post hoc*. *P*-values equal to or below 0.05 were considered significant. A non-parametric Spearman rank correlation test was used to identify relationships between CYP450 protein abundancy and activity for each age category separately, and all ages together. CYP450 proteins with a correlation coefficient equal to or bigger than 0.7 were considered to be involved in the biotransformation of the substrate if more than seven correlations were statistically significant (*p* < 0.05). All analyses were performed in Rstudio (version 1.0.153, RStudio Inc, Boston, MA, United States). Finally, stepwise linear regression analysis was performed on the log-transformed data to evaluate allometric relationships for the intrinsic clearance (biotransformation rate for the full liver) and protein abundance of the involved CYP450 proteins as determined in the correlation analysis. Liver weight, body weight, and sex were the covariates of interest (SPSS 23, IBM, United States).

## Results

### CYP450 Enzyme Activity

**FIGURE 1:**
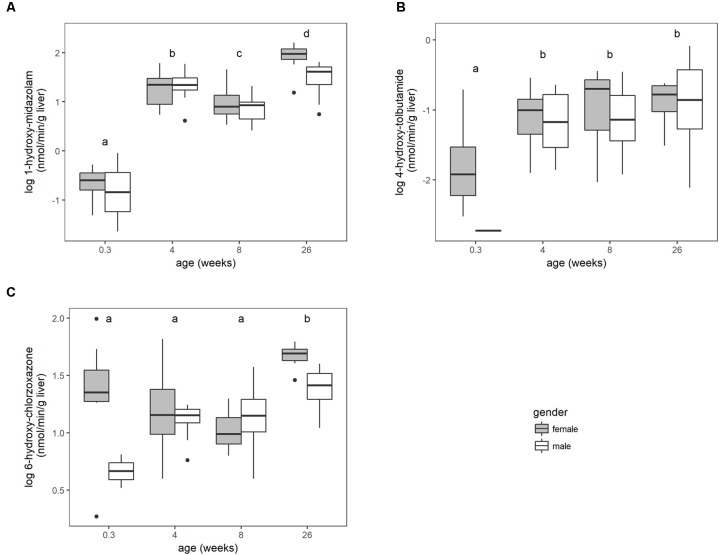
The biotransformation rate of midazolam to 1-hydroxy-midazolam **(A)**, tolbutamide to 4-hydroxy-tolbutamide **(B)** and chlorzoxazone to 6-hydroxy-chlorzoxazone **(C)**, expressed as nmol per min per gram liver of conventional pigs (2-day-old ♂ = 2, ♀ = 8; 4 weeks, 8 weeks, and 6–7 months, each time ♂ = 8, ♀ = 8). No significant sex differences within the age groups were observed. Different letters indicate significant differences (*P* < 0.05) between the different age groups. The boxplots give the median, 25th and 75th percentiles. The upper and lower whisker extends from the hinge to the largest/smallest value, respectively, no further than 1.5 times the interquartile range. Data beyond the end of the whiskers are outliers and plotted individually.

The microsomal enzyme activity (pmol/min/mg microsomal protein) increased with age for the three substrates (Supplementary Figure S1). No significant sex differences were found within the different age groups when the activity was expressed as per mg microsomal protein. This activity was corrected for enzyme losses during the fractionation process according to equation 1. The main effect of gender was significant (*P* < 0.05) for chlorzoxazone when the activity was converted to per g liver. Overall, the biotransformation rate for the three substrates increased with age, but differently according to the enzymes involved (**Figure 1**).

### Microsomal Protein Per Gram Liver

**FIGURE 2:**
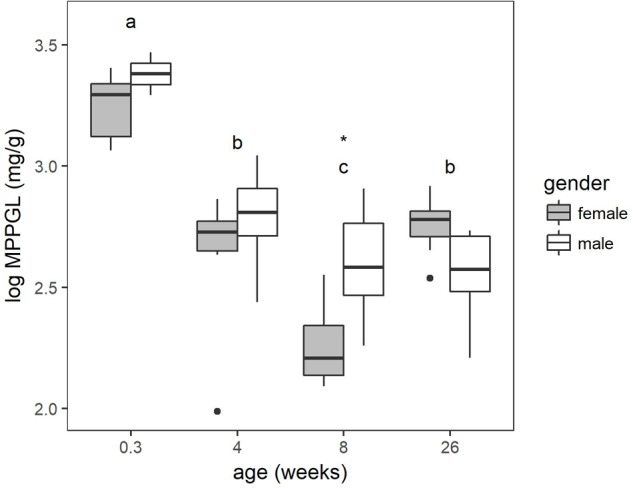
Boxplot of the microsomal protein per gram liver (MPPGL) with increasing age of conventional pigs (2-day-old ♂ = 2, ♀ = 8; 4 weeks, 8 weeks, and 6–7 months, each time ♂ = 8, ♀ = 8). The boxplots give the median, 25th and 75th percentiles. The upper and lower whisker extends from the hinge to the largest/smallest value, respectively, no further than 1.5 times the interquartile range. Different letters indicate significant differences (*P* < 0.05) between the different age groups. The asterisk shows significant sex differences within the age group.

The protein content estimated by the Bradford assay was corrected for protein loss using the microsomal recovery factor. The MPPGL calculated according to equation 3 is depicted in **Figure 2**. The main effect of gender within the age groups was significant (*P* < 0.05) and at 8 weeks of age sex differences were found to be significant. The youngest age category had a significant higher MPPGL compared to the other age categories. Significant differences were also observed between the 4-week-old pigs and the 8-week-old pigs and the 8-week-old pigs and 6- to 7-month-old pigs. No significant differences were observed between the 4-week-old pigs and the 6- to 7-month-old pigs.

### CYP450 Protein Abundancy

**FIGURE 3:**
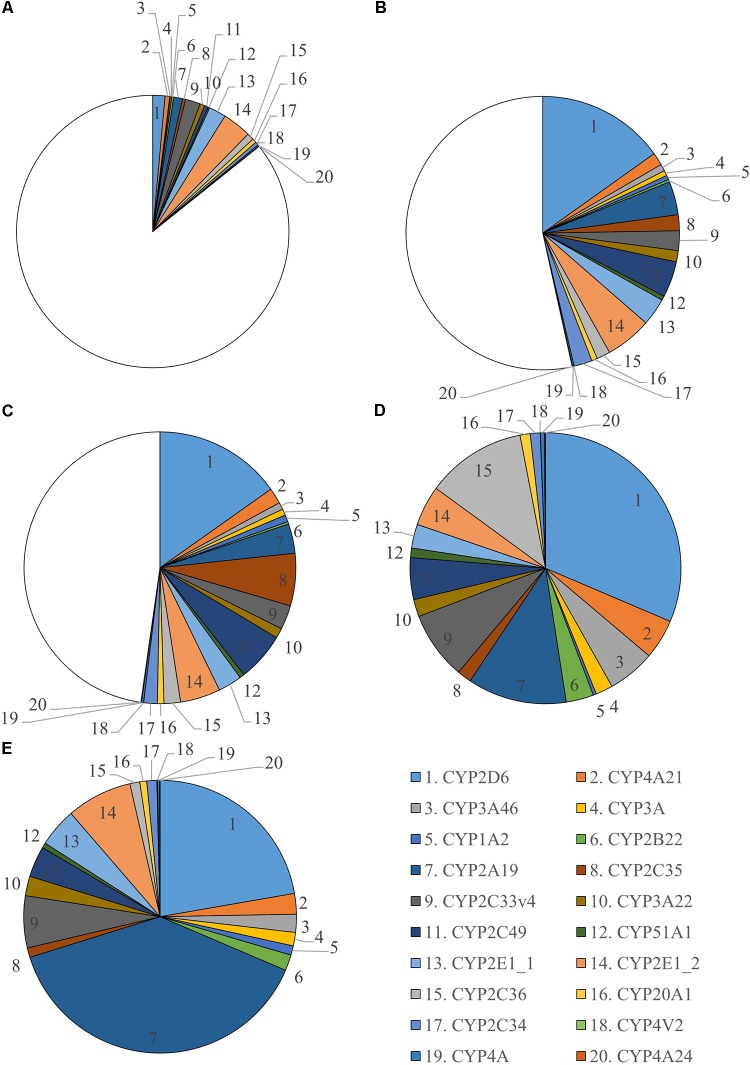
Ontogeny of CYP450 enzymes in growing conventional piglets (2-day-old, 4 weeks, 8 weeks and 6–7 months, each time ♂ = 8, ♀ = 8). Six to seven months old pigs are used as reference and CYP450 abundancy is represented as a full pie of 100%. Since this is the only age category where significant sex differences were found, separate charts for male **(D)** and female **(E)** are shown. For the 2-day- **(A)**, 4-week- **(B)** and 8-week-old **(C)** piglets, the average of both sexes is presented.

In total 255 proteins were retrieved in the liver microsomes. Twenty CYP450 proteins were identified from which 12 had 3 or more unique peptides (Supplementary Table S1). All unique peptides were verified in Mascot. Two different CYP2E1 proteins were found which have a different protein existence. This is shown as a number after an underscore, namely CYP2E1_1 and CYP2E1_2. Number 1 means there is experimental evidence at protein level for the existence of the protein. The criteria include partial or complete Edman sequencing, clear identification by MS, X-ray, or NMR structure, good quality protein–protein interaction or detection of the protein by antibodies. The number 2 means there is evidence of protein existence at transcript level which means that the existence has not been strictly proven but that expression data (such as existence of cDNA(s), RT-PCR or Northern blots) indicate the existence of a transcript. Verification of these two proteins identified small differences in sequence, hence, these enzymes were considered as two different proteins. A relative quantification was performed to evaluate sex and age differences for every CYP450 enzyme found (Supplementary Figure S2). Significant sex differences in abundancy were only found in the 6- to 7-month-old pigs for CYP1A2, CYP2A19, CYP3A22, CYP2E1_1, CYP2E1_2, CYP4V2, and CYP2C36. The absolute quantification against Hi3 *E. coli* peptides was used to visualize the maturation of CYP450 enzymes in microsomes (**Figure 3**). The CYP450 proteins in the 6- to 7-month-old pigs were used as reference and are represented as a full pie of 100%. This abundancy was used to calculate the percentage CYP450 protein of the younger age categories. The individual percentages per age category can be found in Supplementary Table S2. A graphical overview of the absolute amount of CYP450 proteins (fmol) found in microsomes for every age category and per sex can be found in Supplementary Figure S3. In total 133.2 (±7.91), 435.2 (±30.83), 488.5 (±31.89), 683.3 (±47.67), and 1,186.8 (±108.11) pmol CYP450 proteins per mg microsomal protein were determined for 2-day-, 4-week-, 8-week-, 6- and 7-month-old-pigs, respectively.

### Correlation CYP450 Protein Abundance Versus Enzyme Activity

The results of the Spearman rank test can be found in the correlation matrices presented in Supplementary Figure S4. To evaluate which enzyme might be involved in the biotransformation of the probe substrates over all age categories, a correlation coefficient of at least 0.7 was assumed when more than seven CYP450 proteins showed a statistically significant (*P* < 0.05) correlation. The results for each age category, and all ages together can be found in **Table 1**.

**Table 1:** CYP450 proteins showing a statistically significant (*P* < 0.05) Spearman correlation coefficient with the different substrates.

	2-Day-old (*n* = 16)	4-Week-old (*n* = 16)	8-Week-old (*n* = 16)	6- to 7-months-old (*n* = 16)	All pigs (*n* = 64)
Midazolam	CYP4A CYP4A24	/	CYP2C34 CYP2C49	CYP1A2 CYP2A19 CYP3A CYP3A22 CYP2E1 (PE = 1 and PE = 2)	CYP3A46, CYP3A, CYP3A22
Tolbutamide	CYP2C33v4 CYP2C49 CYP2D6	CYP2C49 CYP2C34	CYP2C34 CY^⋅⋅^2C35 CYP2C36 CYP2C49 CYP4V2	CYP2B22 CYP2C34 CYP2C35 CYP2C36 CYP3A CYP51A1	CYP2C35, CYP2C49, CYP2C36, CYP2C34
Chlorzoxazone	CYP2C34	/	CYP2A19	CYP1A2 CYP2A19 CYP3A CYP3A22 CYP2E1 (PE = 1 and PE = 2) CYP4V2	CYP3A46, CYP3A, CYP2C33v4

*Each age category consists of 8 males and 8 females. / No statistically significant correlation coefficient was found. PE, protein existence, these are two different CYP2E1 proteins.*

### Regression Models

**FIGURE 4:**
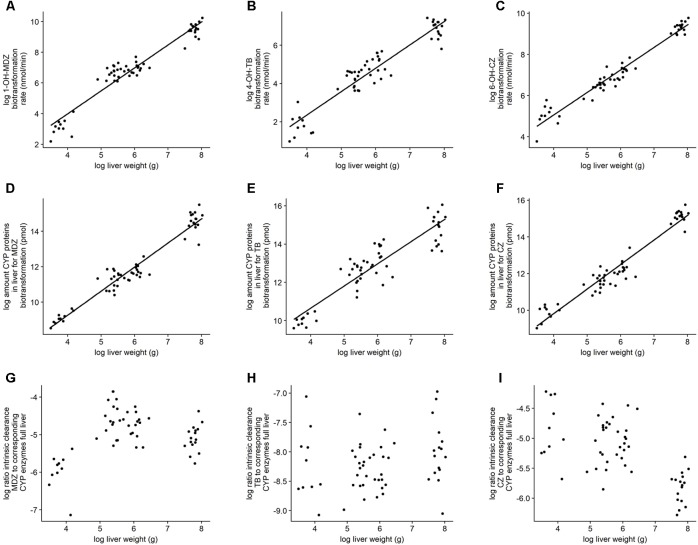
Illustration of the linear regression models. **(A–C)** Bilogarithmic plot of biotransformation rate for the full liver or intrinsic clearance in function of liver weight. **(D–F)** Bilogarithmic plot of amount of CYP450 proteins in full liver involved in the biotransformation of the corresponding probe substrate in function of liver weight. **(G–I)** Bilogarithmic plot of the ratio of the intrinsic clearance to their corresponding amount of CYP450 proteins in function of the liver weight. The graphs are each time for midazolam, tolbutamide, and chlorzoxazone, respectively (left, middle, right).

The log-transformed activity and abundancy data showed a linear relationship which is in support with the principle of allometric scaling. Both intrinsic clearance and protein abundance showed a better fit toward liver weight instead of body weight. Details on the best fits can be found in **Table 2**. **Figure 4** gives an overview of the defined models with the corresponding observations. Additionally, the log ratio of the intrinsic clearance to the corresponding amount of biotransforming CYP450 proteins in the full liver was calculated. No correlation could be observed in function of the log liver weight.

**Table 2:** Properties of the different regression models regarding intrinsic clearance for midazolam, tolbutamide, and chlorzoxazone, the amount of CYP450 proteins in the liver for the biotransformation of the corresponding probe substrate and liver and body weight.

Y = 10^a+b^^∗^ X^c+d^						
Y	X	a (SE)	b (SE)	c (SE)	d (SE)	*R*^2^
LW (g)	BW (kg)	1.48 (0.014)		0.98 (0.015)		0.99
*Female*			-0.04 (0.014)		0.06 (0.015)	
*Male*			0.04 (0.014)		-0.06 (0.015)	
Intr Cl MDZ (nmol/min)	LW (g)	-0.87 (0.15)	ns	1.50 (0.057)	ns	0.92
Intr Cl TB (nmol/min)	LW (g)	-1.10 (0.15)	ns	1.22 (0.055)	ns	0.90
Intr Cl CZ (nmol/min)	LW (g)	0.26 (0.084)		1.11 (0.032)		0.96
*Female*			0.04 (0.019)		ns	
*Male*			-0.04 (0.019)		ns	
CYP450 proteins MDZ (pmol)	LW (g)	1.66 (0.12)	ns	1.36 (0.047)	ns	0.94
CYP450 proteins TB (pmol)	LW (g)	2.61 (0.19)	ns	1.16 (0.07)	ns	0.83
CYP450 proteins CZ (pmol)	LW (g)	1.94 (0.12)	ns	1.34 (0.047)	ns	0.94

*When the effect of gender was significant (P < 0.05) this was included in the equation. LW, liver weight (g); BW, body weight (kg); Intr Cl, intrinsic clearance; MDZ, midazolam; TB, tolbutamide; CZ, chlorzoxazone; CYP450, cytochrome P450; ns, not significant; SE, standard error.*

## Discussion

Despite an increasing amount of data suggesting the piglet as a suitable animal model for pediatric preclinical studies, a lot of knowledge gaps still remain. Especially data on the ontogeny of the different organ systems involved in the ADME processes are lacking. This information is crucial to make reliable predictions of the behavior of a drug in growing children, based on juvenile animal data.

### Microsomal Protein Per Gram Liver

One of the strategies to predict *in vivo* drug clearance in humans, is to extrapolate based on *in vitro* data. The initial step in this process is to convert *in vitro* microsomal enzyme activity expressed as units per milligram protein to per gram whole liver. This conversion is mostly done by a hepatic scaling factor such as the MPPGL. In humans, different adult values of 33, 40, and 52.9 mg/g liver were reported (Lipscomb et al., 2003; Wilson et al., 2003; Hakooz et al., 2006). The differences in adult MPPGL can be due to the liver source, disease state, and/or experimental methodologies. De Bock et al. (2014) measured an average value of 18.73 mg/g in four children with a mean age of 1.17 years suffering from biliary atresia. Barter et al. (2008) suggested increasing MPPGL values from birth (26 mg/g) to a maximum of 40 mg/g at around 28 years of age, followed by a gradual decrease when aging. However, it should be noted that the data from Barter et al. (2008) are based on a limited number of pediatric livers (11 fetal, age not provided; and 5 pediatric, mean age of 7.8 years). In the current study in pigs, the ontogeny of the MPPGL showed a different slope compared to the model of Barter et al. (2008). A decrease from 26.7 mg/g in neonatal piglets to 15.6, 11.7, and 14.4 mg/g at, respectively, 4 and 8 weeks and 2 6–7 months was demonstrated. Only one other study was found that determined the porcine MPPGL. Achour et al. (2011) reported a higher value of 32.6 and 36.2 mg/g liver in two Suffolk White adult pigs. These higher values compared to the values reported in the current study can be due to breed differences. Furthermore, the age was not exactly specified. To the authors knowledge, no study is available where the MPPGL is determined in growing piglets. In order to evaluate whether the porcine MPPGL can be extrapolated to humans, more pediatric data is mandatory in addition to the data provided by Barter et al. (2008).

### CYP450 Enzyme Activity

The biotransformation rate of midazolam in pigs showed a steep increase during the first weeks of life followed by a gradual increase till 6–7 months of age. An increase in midazolam biotransformation rate with age was also observed by Hu (2015) in Camborough-29 pigs and by Van Peer et al. (2017) in Göttingen minipigs. In humans, midazolam is used as a selective probe for CYP3A4/5 enzyme activity. Several human models based on *in vitro* midazolam activity data have been proposed showing the maturation of CYP3A4 with age (Björkman, 2005; Edginton et al., 2006; Johnson et al., 2006). The human maturation curves are in line with the results found in the current study. Edginton et al. (2006) reported an enzyme activity greater than adult values around the first year or two of life. This higher activity is also observed in 4-week-old piglets compared to 8-week-old piglets (**Figure 1**).

The enzyme activity of tolbutamide in pigs, which is a specific human CYP2C9 substrate, increased gradually to a plateau with adult values reached at 8 weeks of age. Hu (2015) noticed a slightly different maturation curve in pigs, where no plateau was reached at 20 weeks of age. In humans, Johnson et al. (2006) reported a similar maturation curve as observed in the current study, where adult values are reached around 6 years of age. It should be mentioned that maturation curves in humans are based on a limited number of pediatric individuals and most liver samples are donated by diseased children, while all experiments in the current study were performed on healthy piglets.

The maturation curve for chlorzoxazone changed drastically after scaling with the MPPGL. This is in contrast to midazolam and tolbutamide, where no differences in maturation were observed whether the activity was expressed as pmol/min/mg protein or nmol/min/g liver. When expressed as pmol/min/mg microsomal protein, the chlorzoxazone activity gradually increased during the first 8 weeks of life, reaching a plateau at 6–7 months of age. When expressed as nmol/min/g liver, the activity remained constant during the first 8 weeks of life and increased when reaching puberty. This demonstrates the importance of a hepatic scaling factor taking the enzyme losses during microsome preparation into account, such as the MPPGL used here. Moreover, Zhang et al. (2015) found the CYP450 activity based on liver tissue to be superior to the CYP450 activity based on microsomal proteins in human. The maturation curve for human CYP2E1, as predicted by Johnson et al. (2006), is different than the maturation curve for the porcine chlorzoxazone biotransformation. This is probably due to the fact that chlorzoxazone is metabolized by more or other porcine enzymes than CYP2E1 (Wiercinska and Squires, 2010).

### CYP450 Protein Abundancy

In order to identify which CYP450 enzyme is involved in the biotransformation of a certain substrate, a correlation analysis between the enzyme abundance in the microsomes and the activity was performed. It is assumed that the more enzyme is present, the higher the transformation rate will be. In literature, protein abundances are mostly determined via mRNA expression (PCR) or immunoquantification (Anzenbacher et al., 1998; Skaanild and Friis, 2008; Rasmussen et al., 2011). Disadvantages of these techniques are that due to posttranslational modifications, the amount of protein is not strictly regulated by the expression of mRNA. Also, the human or rat antibodies used for immunoquantification are prone to cross-selectivity. Furthermore, a selection of CYP450 enzymes of interest has to be made prior to the experiments. The latter is in contrast with the proteomics approach used in the current study, where the enzyme activity can be correlated with all CYP450 proteins found in the microsomes. Overall for midazolam, it was shown that CYP3A46, CYP3A22, and CYP3A are involved in the biotransformation to 1-hydroxy-midazolam when all pigs of all age categories were analyzed together. These CYP450 show a high percentage of identity with their equivalent human CYP450 enzymes, CYP3A4/5/7/43 (Supplementary Table S1). Midazolam could thus be considered as a selective porcine substrate to evaluate the CYP3A enzyme activity. However, when looking at the individual age categories it seems that only at 6–7 months of age CYP3A22 showed a high correlation with midazolam biotransformation. In the younger age categories other CYP450 proteins seem to be involved in the biotransformation. This might be an indication that during development other CYP450 enzymes take over when the CYP450 of interest is not yet abundant enough. For example, van Waterschoot et al. (2008) observed in CYP3A knockout mice midazolam biotransformation by upregulated CYP2C enzymes. Regarding tolbutamide, CYP2C34, CYP2C35, CYP2C36, and CYP2C49 are involved in its biotransformation when evaluating all age categories together. These porcine CYP450 enzymes have 73.1–80.6% of identity with human CYP2C8/9/18/19, suggesting tolbutamide to be a good substrate to evaluate porcine CYP2C activity. The CYP2C family is involved in the tolbutamide biotransformation from 2-day-old piglets onwards, but at 6–7 months of age it appeared that CYP2B22 and CYP51A1 also contributed to the biotransformation. It was already reported that chlorzoxazone might be a poor substrate to evaluate CYP2E activity in pigs (Skaanild, 2006; Wiercinska and Squires, 2010). This could be confirmed in our study where CYP3A46, CYP3A, and CYP2C33v4 showed the highest correlation with chlorzoxazone biotransformation when all pigs were considered. However, the selectivity of involved CYP450 varied with age. In 2-day-old piglets, CYP2C34 was the CYP450 of interest, while at 8 weeks of age CYP2A19 seemed to be involved in the biotransformation of chlorzoxazone. Finally, at 6–7 months, CYP1A2, CYP2A19, CYP3A, CYP3A22, CYP2E1_1, CYP2E1_2, and CYP4V2 showed a significant correlation. Wiercinska and Squires (2010) found chlorzoxazone to be metabolized by CYP1A1, CYP2E1, CYP2A19, and CYP2C33v4 which is in line with the work presented here. However, it seems that age has an influence on the specific CYP450 involved. The current study also showed a significant correlation between CYP3A22, CYP3A46, and CYP3A on the one hand, and chlorzoxazone on the other hand. This is in contrast with Wiercinska and Squires (2010) were no evidence of chlorzoxazone biotransformation by CYP3A was found. The CYP3A involvement might be due to the high correlation between CYP450 proteins mutually. A high correlation coefficient doesn’t necessarily means that the CYP450 of interest is also involved in the corresponding biotransformation. Ideally, all selected CYP450 proteins should be incubated individually with the substrates to evaluate their contribution. Nevertheless, the use of chlorzoxazone as a porcine CYP2E substrate is not advised. It should be noted that at 4 weeks of age no significant correlations could be found for midazolam or chlorzoxazone. CYP3A22 and CYP3A46 had the highest coefficient of 0.47 and 0.35, respectively, with midazolam. Chlorzoxazone showed the best correlation with CYP2A19, CYP4A, and CYP4A24 with a coefficient of 0.41, 0.46, and 0.46, respectively. Further research is required to evaluate the age-related involvement of different CYP450 proteins in the biotransformation of the corresponding substrates. Recently, in our research group, Schelstraete et al. (2018) included two more probe substrates to gain more insight in the selectivity of porcine CYP450 enzymes (submitted).

There is ample evidence available that the increase in biotransformation rate can be attributed to developmental expression of CYP450 mRNA and proteins in humans and pigs (Alcorn and McNamara, 2002; Van Peer et al., 2015) The current study supports this hypothesis as an increase in biotransformation rate with age can be related to an increase in absolute amount of CYP450 proteins in the microsomes. At 2 days of age only 14% of CYP450 proteins are present compared to the 6- to 7-month-old pigs, which is reflected in a very low biotransformation rate of the three substrates. Four- and eight-week-old piglets have around 50% of CYP450 proteins compared to the 6- to 7-month-old pigs. At 6–7 months of age, the amount of CYP450 proteins is considered to be at maximum adult values with 100% CYP450 proteins present in the liver. Achour et al. (2011) was able to identify 10 CYP450 proteins in microsomes of two Suffolk white adult pigs. Both CYP2D6 and CYP2A19 were most abundant, which is in line with the results from the current study with an abundancy of 31 and 12% for males and 22 and 39% for females for CYP2D6 and CYP2A19, respectively. Achour et al. (2011) did not report any sex differences, as the study was only done in two pigs. Significant sex differences in activity and mRNA expression for CYP1A, CYP1A2, and CYP2A were observed by Rasmussen et al. (2011) in intact crossbreeds between Landrace × Yorkshire sire and Duroc boars of 164 days old. The CYP2E1 activity was higher in females compared to males, although not significant. Despite the higher activity and mRNA expression in female pigs, Rasmussen et al. (2011) did not found elevated levels of protein with western blotting in females. This is in contrast with the results presented here, where the 7 months old females have significantly higher CYP1A2, CYP2A19, CYP3A22, CYP4V2, CYP2E1_1, and CYP2E1_2 protein abundances. Higher female protein concentrations were also observed by Skaanild and Friis (1999) for CYP1A2 and CYP2E1 in both Göttingen minipigs and Danish Landrace × Yorkshire × Duroc cross-breed pigs (Skaanild and Friis, 1999). The 6-month-old males in the current study showed significant higher CYP2C36 protein abundance compared to females. The observed sex differences could be attributed to sex hormones. Van den Broeke et al. (2015) observed in 264 conventional pigs (Hybrid sow × Piétrain boar) that at 5 months of age, all boars reached puberty, but only 8–31% of gilts were in puberty. Therefore, in this study, gilts of 7 months old were included to represent sows in puberty. In order to evaluate whether these sex differences in CYP450 protein abundance have an influence on the biotransformation of drugs, selective substrates should be chosen to perform the same activity measurements as described above for midazolam, tolbutamide, and chlorzoxazone. In the current study, no significant sex differences in enzyme activity were found for these substrates. Although, this could have been expected for tolbutamide as CYP2C36 is involved in its biotransformation. This could possibly mean that other CYP450 enzymes contributed more to the biotransformation of tolbutamide than CYP2C36.

In humans, several studies have been performed to determine the protein abundance of the CYP450 enzymes in adult microsomes. This resulted in great variations of reported values. For example, Gröer et al. (2014) reported CYP3A4 abundance of 32.6 pmol/mg microsomal protein while Achour et al. (2017) measured 80.9 pmol/mg microsomal protein. A possible explanation could be the different methods used, rendering it difficult to compare the absolute porcine abundances with human. A better way to make a correlation between these two species is to compare the relative abundances. CYP2D6 (26%) and CYP2A19 (29%) are the most abundant CYP450 proteins in 6- to 7-month-old pigs, followed by CYP2E (13%) and CYP2C (18%) proteins. In humans, however, CYP2C9 (20%) is found to be the most prominent CYP450 protein in microsomes, followed by CYP3A4 (16%), CYP2E1 (13%), and CYP2A6 (12%) (Kawakami et al., 2011). Unfortunately, data regarding the ontogeny of the human CYP450 proteome is limited. One study reported increasing levels of CYP2B6 with age measured using immunoquantification (Pearce et al., 2016). Sadler et al. (2016) performed a more thorough comparison of CYP450 protein expression and activity profiles through human development using activity-based protein profiling. The porcine homologs of the human CYP450 enzymes marked by a postnatal increase, namely members of the CYP1A, 2A, 3A, 2C, 2D, and 2E subfamilies, also increased in protein amount in the current study. Sadler et al. (2016) however, did not found a direct correlation between CYP450 activity and protein expression. This is in contrast with the results presented here where high correlation coefficients were found between the biotransformation rate and certain CYP450 proteins. Further research is required to evaluate whether pediatric microsomes show a similar trend as seen in piglets, namely an increase in amount of CYP450 proteins which can be correlated with an increase in activity.

The biotransformation rate, as well as the amount of involved CYP450 proteins showed a log-linear relationship with the log of the liver weight (*R*^2^ ≥ 0.83) (**Figure 4**). In addition, the log-ratio of the biotransformation rate to their corresponding CYP450 proteins showed no linear correlation in function of the log liver weight. This indicates that when the CYP450 protein is present, it will contribute to the biotransformation of the probe substrates regardless of the age or liver weight of the animal. The developmental biotransformation rates observed in the present study might thus be solely due to an increase in absolute amount of CYP450 proteins. Further research is, however, required to unravel the mechanisms regulating CYP450 enzyme ontogeny.

## Conclusion

The ontogeny of porcine CYP450 enzymes was established on three levels, i.e., enzyme activity with and without hepatic scaling factor and protein abundance. The increase in biotransformation rate with age could be attributed to an increase in absolute amount of CYP450 proteins. Midazolam and tolbutamide were shown to be selective probe substrates to evaluate both human and porcine CYP3A and CYP2C enzyme activities, respectively. Chlorzoxazone is not advised to use as a selective porcine CYP2E substrate. This study provides more insight in the ontogeny of CYP450 mediated phase I metabolism in pigs, which can be used to support pediatric preclinical research. Further research is required to evaluate age-related changes in CYP450 biotransformation involvement and the regulating developmental mechanisms. Also, other relevant CYP450 enzymes and substrates should be evaluated.

## Data Availability Statement

The raw data supporting the conclusions of this manuscript will be made available by the authors, without undue reservation, to any qualified researcher.

## Author Contributions

JM, EGa, LDB, JVB, DD, SC, MD, EGo, and LDC contributed conception and design of the study. JM organized the database and performed statistical analysis. SS performed linear regression analysis. JM, EGa, WS, EGo, and LDC aided in the acquisition of the work. JM wrote the first draft of the manuscript. EGo and LDC wrote a section of the manuscript. All authors contributed to manuscript revision, read and approved the submitted version.

## Conflict of Interest Statement

The authors declare that the research was conducted in the absence of any commercial or financial relationships that could be construed as a potential conflict of interest. The reviewer JH and handling Editor declared their shared affiliation.

## Footnotes

1 http://www.thegpm.org/crap/

## Abbreviations

ACN: acetonitrile
ADME: absorption, distribution, metabolism, and excretion
CaCl_2_: calcium chloride
CID: collision induced dissociation
CYP450: cytochrome P450
DMSO: dimethyl sulfoxide
DTT: dithiothreitol
EMA: European Medicines Agency
ESI: electrospray ionization
FDA: Food and Drug Administration
HD-DDA: high definition-data dependent analysis
ICH: International Conference on Harmonization of Technical Requirements for Registration of Pharmaceuticals for Human Use
IVIVE: *In vitro* to *in vivo* extrapolation
KCl: potassium chloride
KCN: potassium cyanide
K_M_: Michaelis-Menten constant
MMTS: methyl methanethiosulfonate
MPPGL: microsomal protein per gram liver
MS: mass spectrometry
NADPH: β-nicotinamide adenine dinucleotide phosphate
NCR: NADPH-cytochrome *c* reductase
PD: pharmacodynamics
PK: pharmacokinetics
TEABC: triethylammonium bicarbonate buffer
UHPLC-MS/MS: ultra-high performance liquid chromatography tandem mass spectrometry
UPLC: ultra-performance liquid chromatography
V_max_: maximum rate
