# Supplementary material for: The Ontogeny of Cytochrome P450 Enzyme Activity and Protein Abundance in Conventional Pigs in Support of Preclinical Pediatric Drug Research
Source: Front Pharmacol. 2018 May 14;9:470. doi: 10.3389/fphar.2018.00470 (PMC5960725; doi:10.3389/fphar.2018.00470)
Supplement: Supplementary file 1 [file Data_Sheet_1.docx]

**Table S1**: Overview of the identified porcine CYP450 enzymes in hepatic microsomes of conventional pigs (2-day-old, 4 weeks, 8 weeks and 6-7 months old pigs, each time ♂ = 8, ♀ = 8), their accession number, peptide count and the number of unique peptides alongside their equivalent human CYP450 enzyme. The alignment was performed using the BLAST function in Uniprot. If more human CYP450 enzymes showed a high percentage of identity, only the reviewed human proteins were displayed.

| Porcine CYP450 enzyme | Accession number | Peptide count | Unique peptides | Equivalent human CYP450 enzyme | % Identity |
| --- | --- | --- | --- | --- | --- |
| CYP2D6 | A0A0H4ITY7;Q1HLR0 | 26 | 25 | CYP2D6 | 78.9 |
|  |  |  |  | CYP2D7 | 73.4 |
|  |  |  |  | CYP2D6 isoform 2 | 70.3 |
| CYP3A22 | A0A1B2TT60 | 12 | 9 | CYP3A4 | 75.7 |
|  |  |  |  | CYP3A5 | 73.9 |
|  |  |  |  | CYP3A7 | 71.8 |
|  |  |  |  | CYP3A43 | 68.1 |
| CYP3A46 | A7KZR2 | 8 | 3 | CYP3A4 | 76.7 |
|  |  |  |  | CYP3A5 | 74.9 |
|  |  |  |  | CYP3A7 | 72.2 |
|  |  |  |  | CYP3A43 | 69.1 |
| CYP3A | B5LX22 | 10 | 4 | CYP3A4 | 76.5 |
|  |  |  |  | CYP3A5 | 75.7 |
|  |  |  |  | CYP3A7 | 72.8 |
|  |  |  |  | CYP3A43 | 68.3 |
| CYP1A2 | F1SJ26 | 7 | 7 | CYP1A2 isoform 2 | 81.6 |
|  |  |  |  | CYP1A2 | 81.4 |
|  |  |  |  | CYP1A1 | 70.5 |
| CYP2B22 | Q8SQ67 | 5 | 4 | CYP2B6 | 74.7 |
|  |  |  |  | CYP2A13 | 57.1 |
|  |  |  |  | CYP2A6 | 55.4 |
| CYP2A19 | Q8SQ68 | 22 | 18 | CYP2A13 | 90.3 |
|  |  |  |  | CYP2A6 | 87.7 |
|  |  |  |  | CYP2A7 | 85.2 |
| CYP2C34 | Q5S8C4 | 7 | 3 | CYP2C18 | 78.4 |
|  |  |  |  | CYP2C9 | 77.1 |
|  |  |  |  | CYP2C19 | 75.5 |
|  |  |  |  | CYP2C8 | 75.0 |
| CYP2C35 | Q28979 | 4 | 1 | CYP2C18 | 75.8 |
|  |  |  |  | CYP2C8 | 74.4 |
|  |  |  |  | CYP2C19 | 73.6 |
|  |  |  |  | CYP2C9 | 74.0 |
| CYP2C36 | Q28980 | 3 | 1 | CYP2C18 | 73.6 |
|  |  |  |  | CYP2C19 | 72.2 |
|  |  |  |  | CYP2C8 | 73.6 |
|  |  |  |  | CYP2C9 | 73.1 |
| CYP2C33v4 | Q8SQ66 | 22 | 19 | CYP2C9 | 63.3 |
|  |  |  |  | CYP2C19 | 62.9 |
|  |  |  |  | CYP2C18 | 61.6 |
|  |  |  |  | CYP2C8 | 62.1 |
| CYP2C49 | F1SC62 | 14 | 8 | CYP2C18 | 80.6 |
|  |  |  |  | CYP2C9 | 78.6 |
|  |  |  |  | CYP2C19 | 77.8 |
|  |  |  |  | CYP2C8 | 75.9 |
| CYP51A1 | D0G6S0 | 4 | 4 | Lanosterol 14-alpha demethylase | 95.2 |
|  |  |  |  | Lanosterol 14-alpha demethylase isoform 2 | 97.3 |
| CYP2E1 (PE=1) | F1SCT4 | 15 | 1 | CYP2E1 | 79.5 |
|  |  |  |  | CYP2C18 | 56.9 |
|  |  |  |  | CYP2C19 | 56.1 |
|  |  |  |  | CYP2C9 | 56.1 |
| CYP2E1 (PE=2) | Q8SQ64 | 15 | 1 | CYP2E1 | 79.5 |
|  |  |  |  | CYP2C18 | 57.1 |
|  |  |  |  | CYP2C19 | 56.1 |
|  |  |  |  | CYP2C9 | 56.1 |
| CYP20A1 | A0A0H4IRA9 | 1 | 1 | CYP20A1 | 91.6 |
|  |  |  |  | CYP20A1 isoform 2 | 82.4 |
| CYP4A | Q95JF8 | 10 | 1 | CYP4A11 | 75.6 |
|  |  |  |  | CYP4A22 | 74.1 |
| CYP4A24 | CP4AO | 10 | 1 | CYP4A11 | 75.6 |
|  |  |  |  | CYP4A22 | 74.3 |
| CYP4A21 | A0A0H4J4B0 | 14 | 6 | CYP4A11 | 75.6 |
|  |  |  |  | CYP4A22 | 73.9 |
| CYP4V2 | A7KZS1 | 1 | 1 | CYP4V2 | 77.4 |
|  |  |  |  | CYP4V2 isoform 2 | 77.4 |
| PE: Protein existence; PE=1 : experimental evidence at protein level; PE=2 : experimental evidence at transcript level.  The different PE is annotated after an underscore, so further on CYP2E1_1 and CYP2E_2 is used. | | | | | |

1. (b)


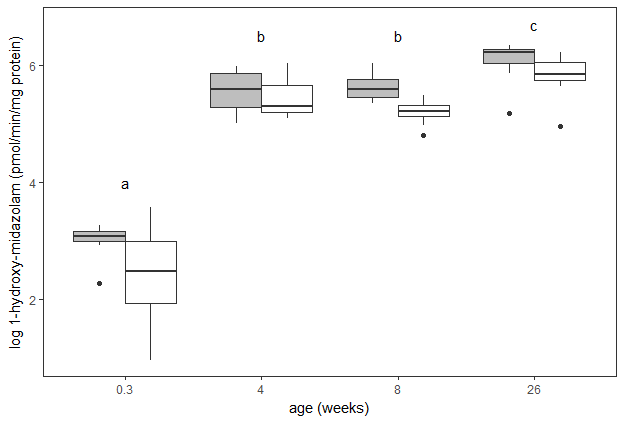

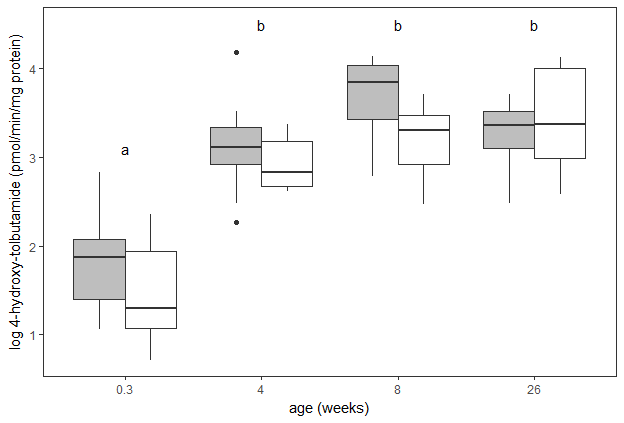


(c)


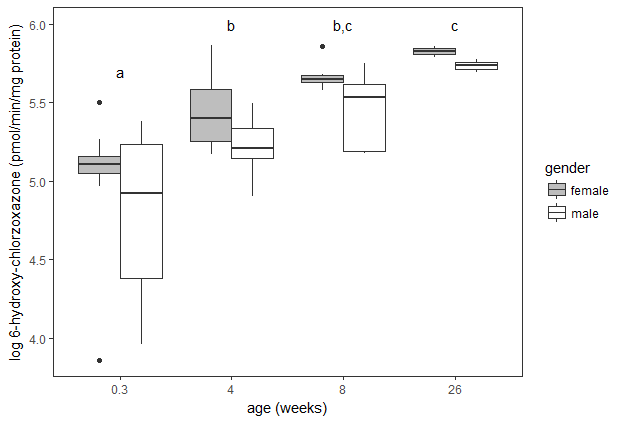


**Figure S1**: The biotransformation rate expressed as pmol/min/mg microsomal protein in hepatic microsomes of conventional pigs (2-day-old, 4 weeks, 8 weeks and 6-7 months, each time ♂ = 8, ♀ = 8) of midazolam to 1-hydroxy-midazolam (a), tolbutamide to 4-hydroxy-tolbutamide (b) and chlorzoxazone to 6-hydroxy-chlorzoxazone (c). The boxplots give the median, 25^th^ and 75^th^ percentiles. The upper and lower whisker extends from the hinge to the largest/smallest value respectively no further than 1.5 times the interquartile range. Data beyond the end of the whiskers are outliers and plotted individually. No significant sex differences were observed. Different letters indicate significant differences between age categories (P < 0.05).


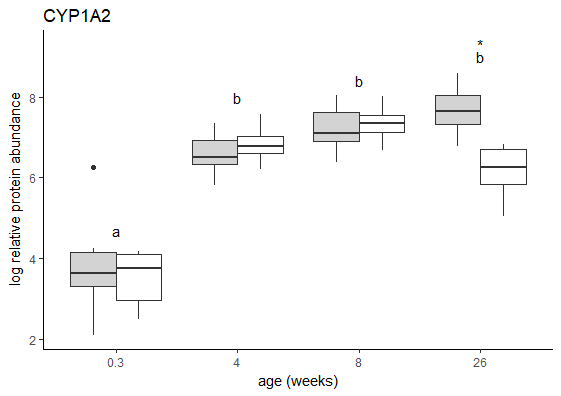

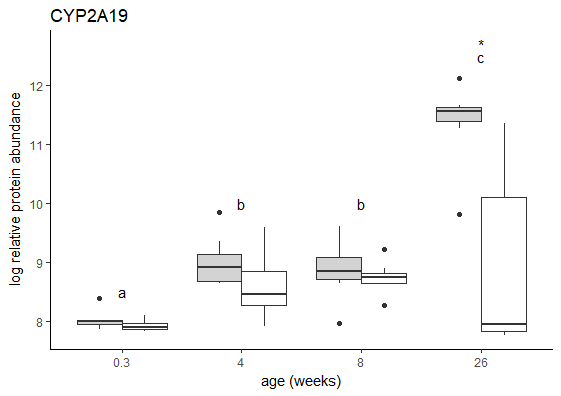

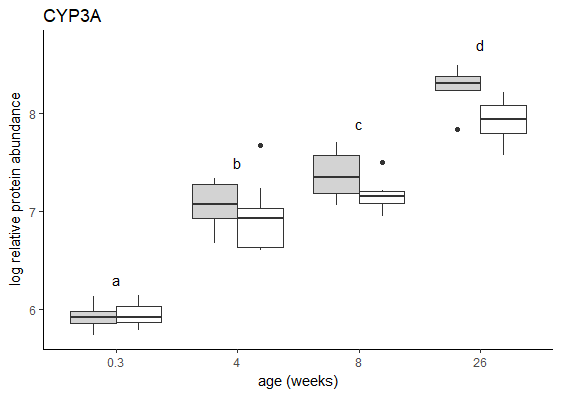

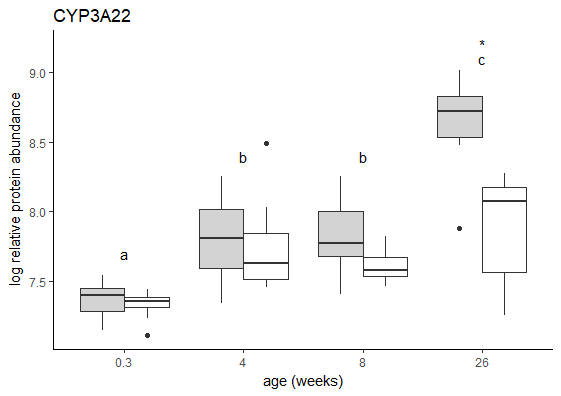

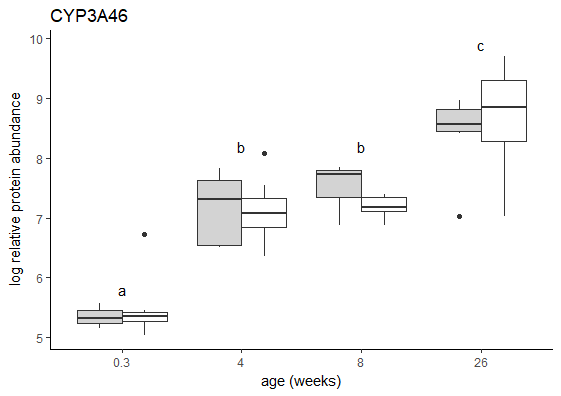

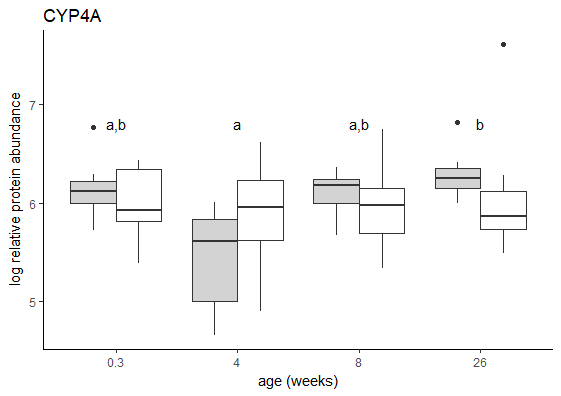

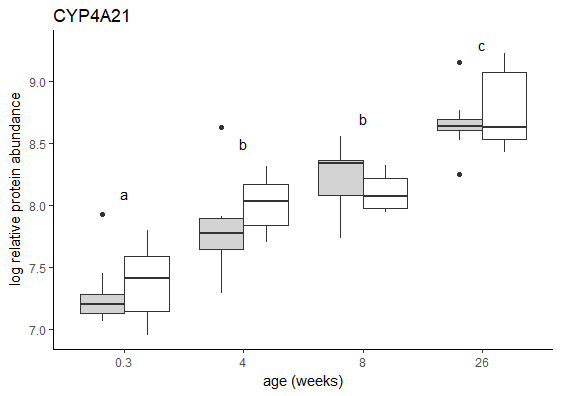

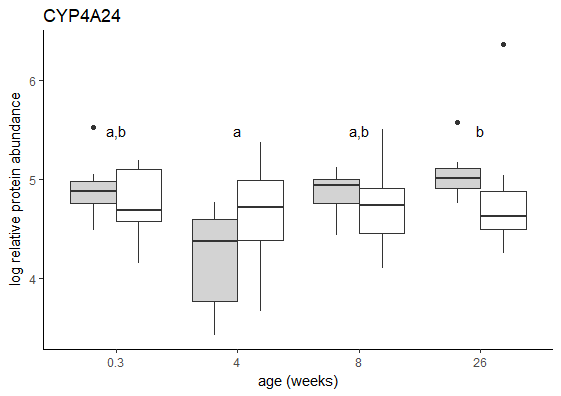

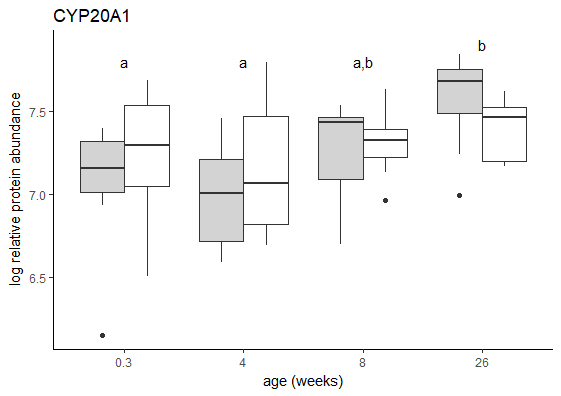

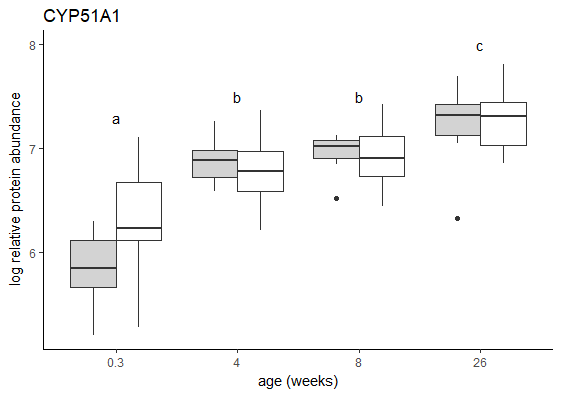

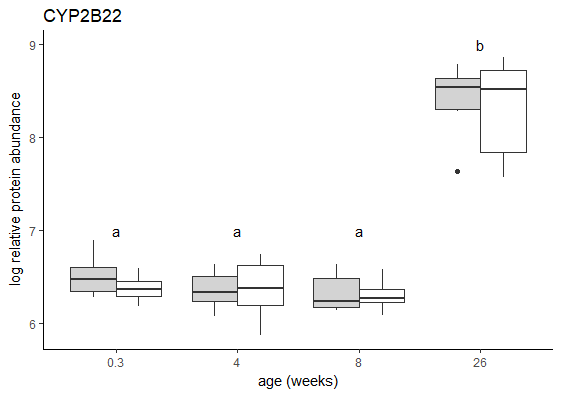

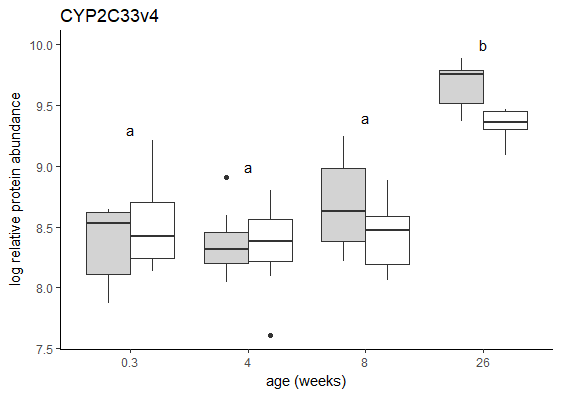

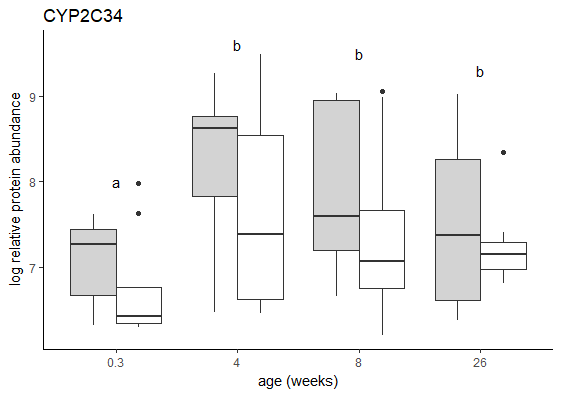

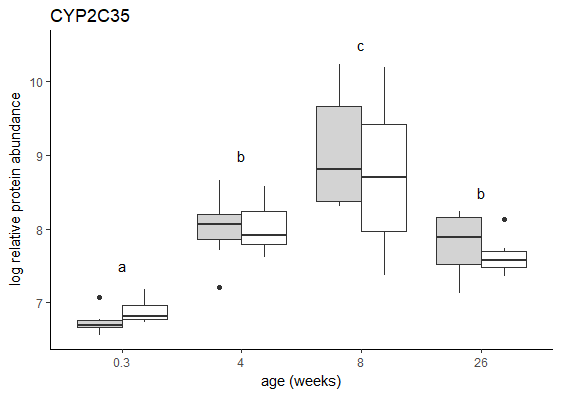

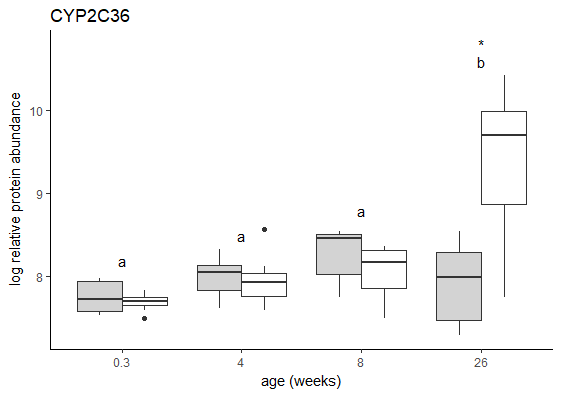

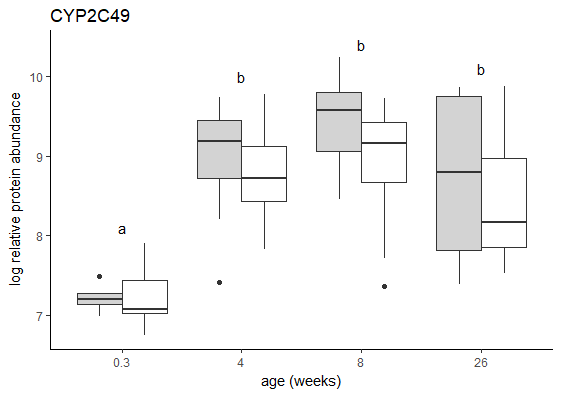

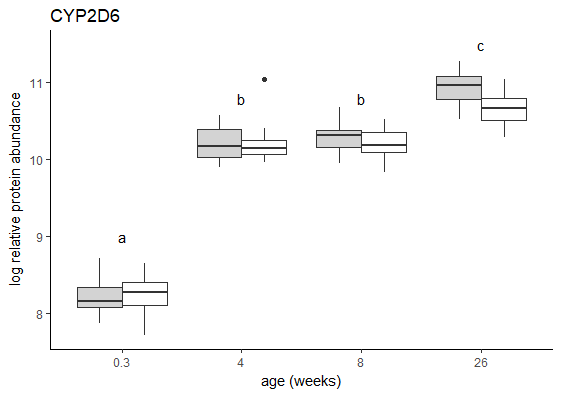

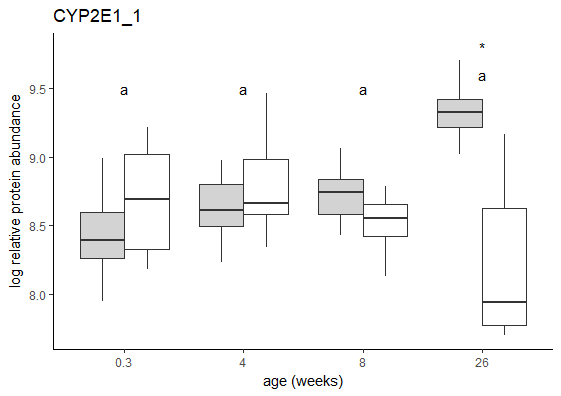

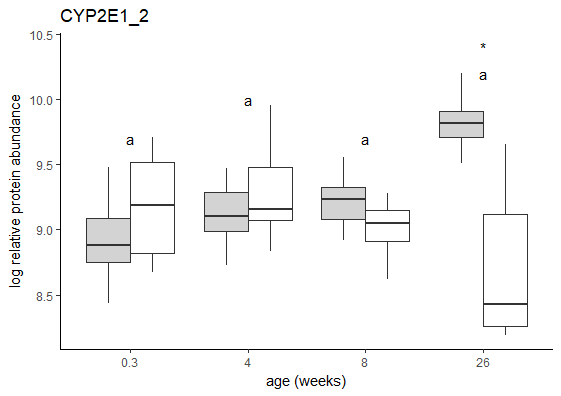

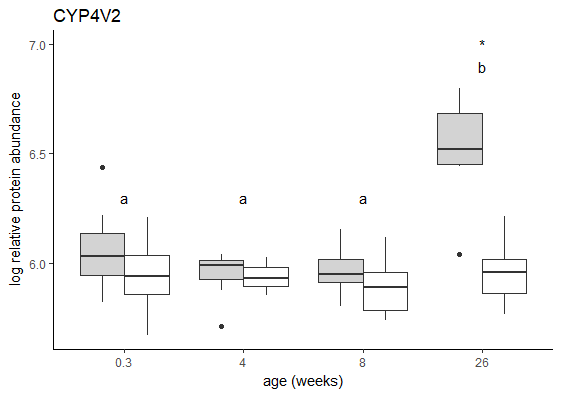


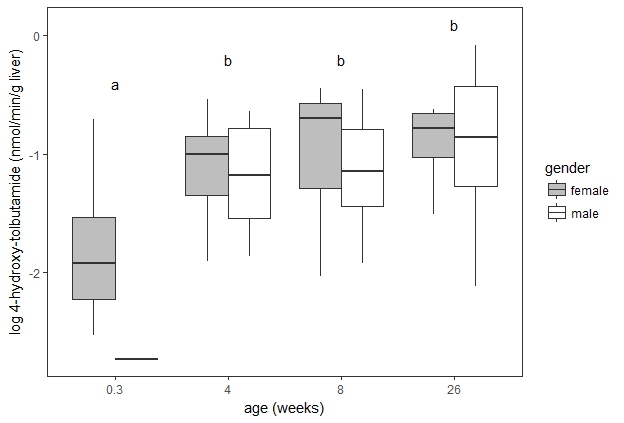


**Figure S2**: Relative protein abundance per CYP450 enzyme identified in hepatic microsomes of conventional pigs (2-day-old, 4 weeks, 8 weeks and 6-7 months old, each time ♂ = 8, ♀ = 8). The boxplots give the median, 25^th^ and 75^th^ percentiles. The upper and lower whisker extends from the hinge to the largest/smallest value respectively no further than 1.5 times the interquartile range. Data beyond the end of the whiskers are outliers and plotted individually. The females are indicated in grey, the males are white. Significant sex differences within the age groups are presented with an asterisk, significant differences in protein abundance across the ages are indicated with a different letter (P < 0.05).

**Figure S3**: Bar chart of the absolute amount of CYP450 proteins found in hepatic microsomes of conventional pigs (2-day-old, 4 weeks, 8 weeks and 6-7 months old, each time ♂ = 8, ♀ = 8). The average of each group is shown with the corresponding standard deviation.

(A)


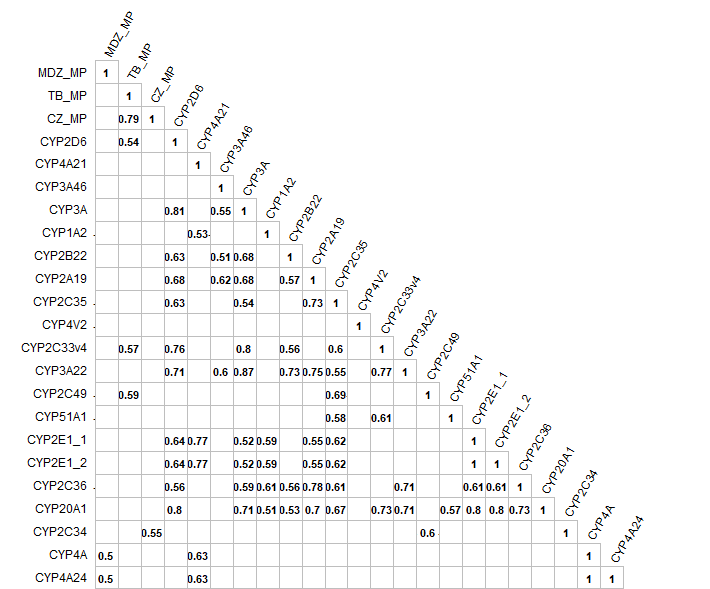


(B)


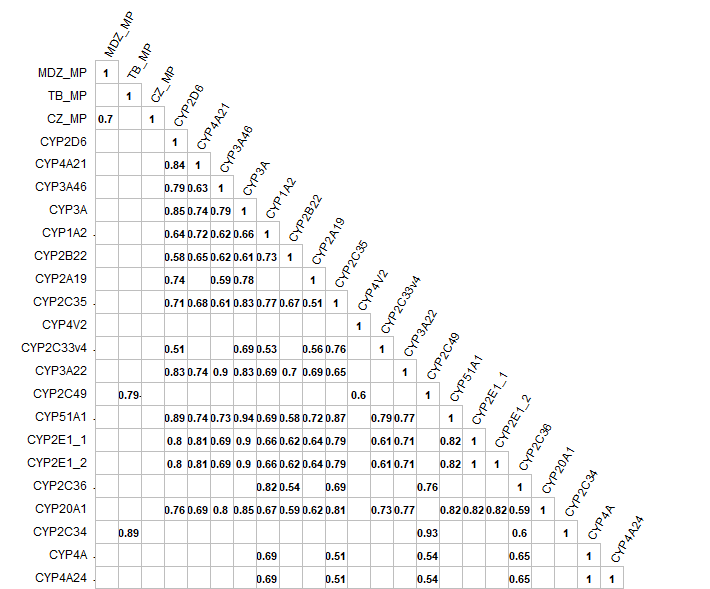


(C)


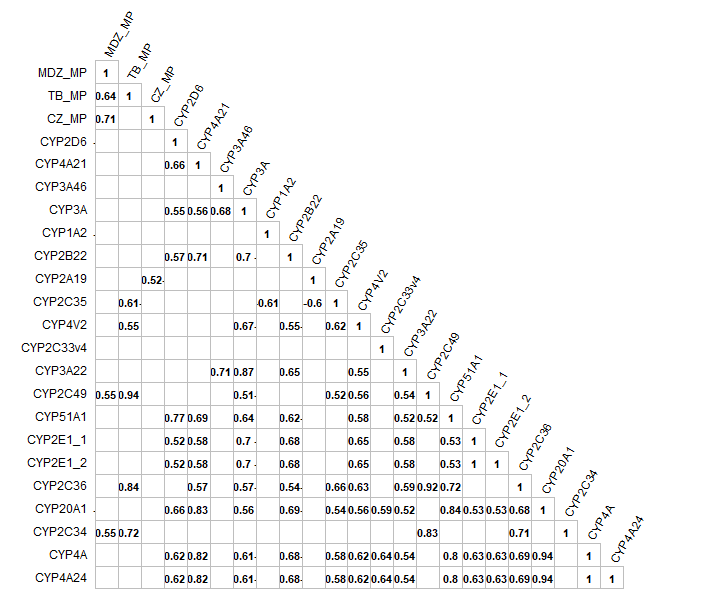


(D)


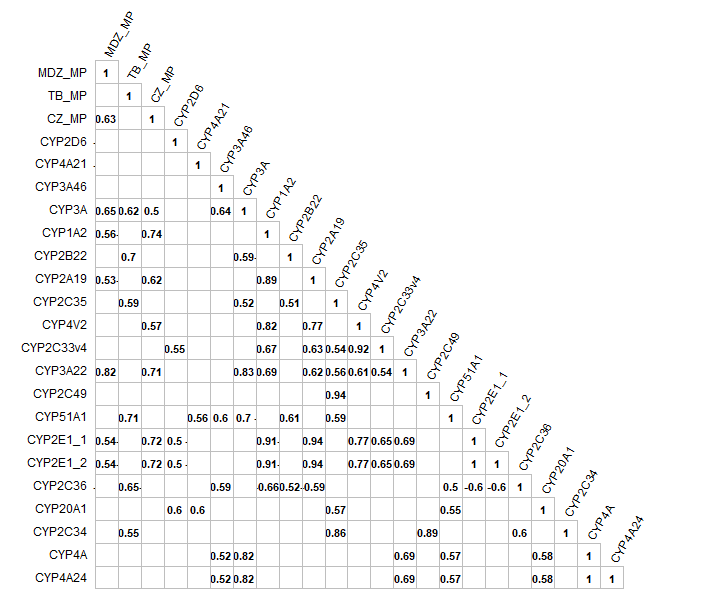


(E)


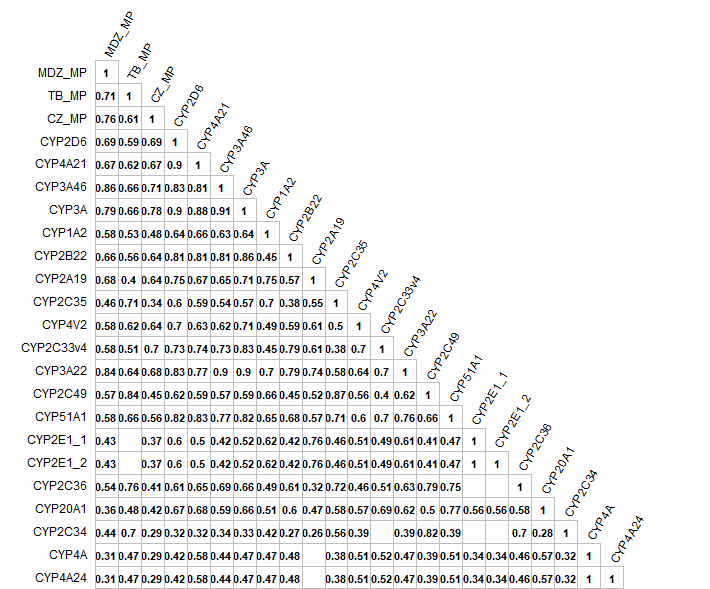


**Figure S4**: Spearman correlation matrix of the different probe substrate activities and CYP450 enzyme proteins found in hepatic microsomes of conventional pigs (2-day-old, 4 weeks, 8 weeks and 6-7 months old, each time ♂ = 8, ♀ = 8). (A-D) Correlation matrices for the 2-day-, 4-week-, 8-week- and 6-7-months-old pigs respectively. (E) Correlation matrix for all pigs across all ages together. MDZ_MP: biotransformation of midazolam to 1-hydroxy-midazolam expressed as pmol/min/mg microsomal protein; TB_MP: biotransformation of tolbutamide to 4-hydroxy-tolbutamide expressed as pmol/min/mg microsomal protein; CZ-MP: biotransformation of chlorzoxazone to 6-hydroxy-chlorzoxazone expressed as pmol/min/mg microsomal protein.

**Table S2**: Percentage of CYP450 enzymes present in hepatic microsomes of conventional pigs (2-day-old, 4 weeks, 8 weeks and 6-7 months old, each time ♂ = 8, ♀ = 8), complementary to the pie charts in figure 3.

| % adult CYP450 | 2 days | 4 weeks | 8 weeks | 6 months male | 7 months female |
| --- | --- | --- | --- | --- | --- |
| CYP2D6 | 1.45 | 15.14 | 15.19 | 31.41 | 22.26 |
| CYP4A21 | 0.55 | 1.50 | 1.87 | 4.76 | 2.45 |
| CYP4V2 | 0.00 | 0.01 | 0.01 | 0.02 | 0.08 |
| CYP4A | 0.16 | 0.18 | 0.24 | 0.47 | 0.22 |
| CYP4A24 | 0.05 | 0.06 | 0.07 | 0.14 | 0.07 |
| CYP3A46 | 0.09 | 0.81 | 0.85 | 5.67 | 2.14 |
| CYP3A | 0.14 | 0.62 | 0.75 | 2.05 | 1.59 |
| CYP3A22 | 0.55 | 1.33 | 1.18 | 2.04 | 2.31 |
| CYP1A2 | 0.02 | 0.52 | 0.82 | 0.37 | 1.11 |
| CYP2A19 | 1.05 | 3.95 | 3.51 | 11.85 | 38.76 |
| CYP51A1 | 0.18 | 0.52 | 0.57 | 1.14 | 0.61 |
| CYP20A1 | 0.50 | 0.67 | 0.79 | 1.21 | 0.83 |
| CYP2B22 | 0.23 | 0.30 | 0.29 | 3.28 | 1.88 |
| CYP2C34 | 0.40 | 2.16 | 1.61 | 1.18 | 1.19 |
| CYP2C35 | 0.35 | 1.89 | 6.24 | 1.69 | 1.13 |
| CYP2C36 | 0.82 | 1.60 | 1.95 | 11.94 | 1.16 |
| CYP2C49 | 0.47 | 4.25 | 5.81 | 4.99 | 3.66 |
| CYP2C33v4 | 1.80 | 2.33 | 2.88 | 8.14 | 6.16 |
| CYP2E1_1 | 2.03 | 3.24 | 2.83 | 2.84 | 4.60 |
| CYP2E1_2 | 3.43 | 5.48 | 4.77 | 4.80 | 7.77 |
| % total CYP450 | 14.25 | 46.55 | 52.25 | 100.00 | 100.00 |
